# Teaching breast ultrasound skills including core-needle biopsies on a phantom enhances undergraduate student’s knowledge and learning satisfaction

**DOI:** 10.1007/s00404-021-06016-8

**Published:** 2021-03-16

**Authors:** G. Schmidt, C. Gerlinger, J. Endrikat, L. Gabriel, C. Müller, S. Baus, T. Volk, Sebastian Findeklee, E. F. Solomayer, A. Hamza, R. Ströder

**Affiliations:** 1 Department of Gynecology, Obstetrics and Reproductive Medicine, University Medical School of Saarland, Kirrberger Straße, 66421 HomburgSaar, Germany; 2 MVZ Fertility Center Hamburg, 20095 Hamburg, Germany; 3 Department of Gynecology, Obstetrics, Kantonsspital Baden AG, 5404 Baden, Switzerland

**Keywords:** Breast ultrasound, Teaching, Undergraduate, Peyton, Core-needle biopsy

## Abstract

**Purpose:**

To investigate whether a training program on breast ultrasound skills including core-needle biopsies to undergraduate students can improve medical knowledge and learning satisfaction.

**Methods:**

Medical students attending mandatory classes at the Medical School of the University of Saarland received a supplemental theoretical and hands-on training program on ultrasound (US) breast screening and on US-guided core-needle biopsy using an agar–agar phantom. Experienced breast specialists and ultrasound examiners served as trainers applying Peyton’s 4-step training approach. The students’ theoretical knowledge and hands-on skills were tested before and after the training program, using a multiple-choice questionnaire (MCQ), the Objective Structured Clinical Examination (OSCE) and a student curriculum evaluation.

**Results:**

The MCQ results showed a significant increase of the student’s theoretical knowledge (50.2–75.2%, *p* < 0.001). After the course, the OSCE showed a mean total of 17.3/20 points (86.5%), confirming the practical implementation of the new skills. The student curriculum evaluation in general was very positive. A total of 16/20 questions were rated between 1.2 and 1.7 (very good) and 3 questions were rated as 2.1 (good).

**Conclusion:**

Undergraduate student’s medical education can be enhanced by teaching breast US skills.

## Introduction

Ultrasound (US) is widely used in diagnostic medicine as it is non-invasive, painless, fast & easy to perform, inexpensive and does not apply ionizing radiation [1]. It provides accurate images of tissue structures and offers valuable diagnostic information. US is used in almost every sub-specialty of obstetrics and gynecology, e.g., fetal medicine, general and specialized gynecology and senology [2, 3].

While US devices are widely used the quality of the devices and the experience of the examiners vary widely. Both factors are key for accurate diagnosis. The examiner must be well-trained and have a deep knowledge of anatomy, physiology and pathology in order to be able to correlate US findings with clinical findings [4].

Today, most undergraduate students passively watch US examinations during their practical training, as available time of the experts and the number of patients willing to take part are limited. Therefore, US screening is not usually included in the undergraduate curriculum. Furthermore, US-guided core-needle biopsy is an essential diagnostic tool for the evaluation of breast lesions [5, 6, 7].

In Germany, breast US training and certification is governed by the German Society of Ultrasound in Medicine (DEGUM). US examiners are classified according to their experience, training and certification into three levels [8]. In order to motivate future clinicians, the society included undergraduate education in their training programs [9]. The ability to use US as a diagnostic and interventional tool is an important complement to basic clinical skills [4, 10, 11, 12].

Training on phantoms increases the students’ confidence and mitigates mistakes [13].

The goal of this study was to assess the effectiveness of an extended US breast training program on the knowledge and hands-on skills of undergraduate medical students.

Similar projects were successfully performed in obstetrics [11] and anesthesiology [14].

## Material and methods

The participating students attended the 5th clinical semester. In addition to their mandatory practical training week in gynecology and obstetrics (which is part of the curriculum), this program was offered for particularly interested students. A total of 8–10 students formed a group, which were further divided in two groups for the practical training.

### Training program

**Fig. 1:**
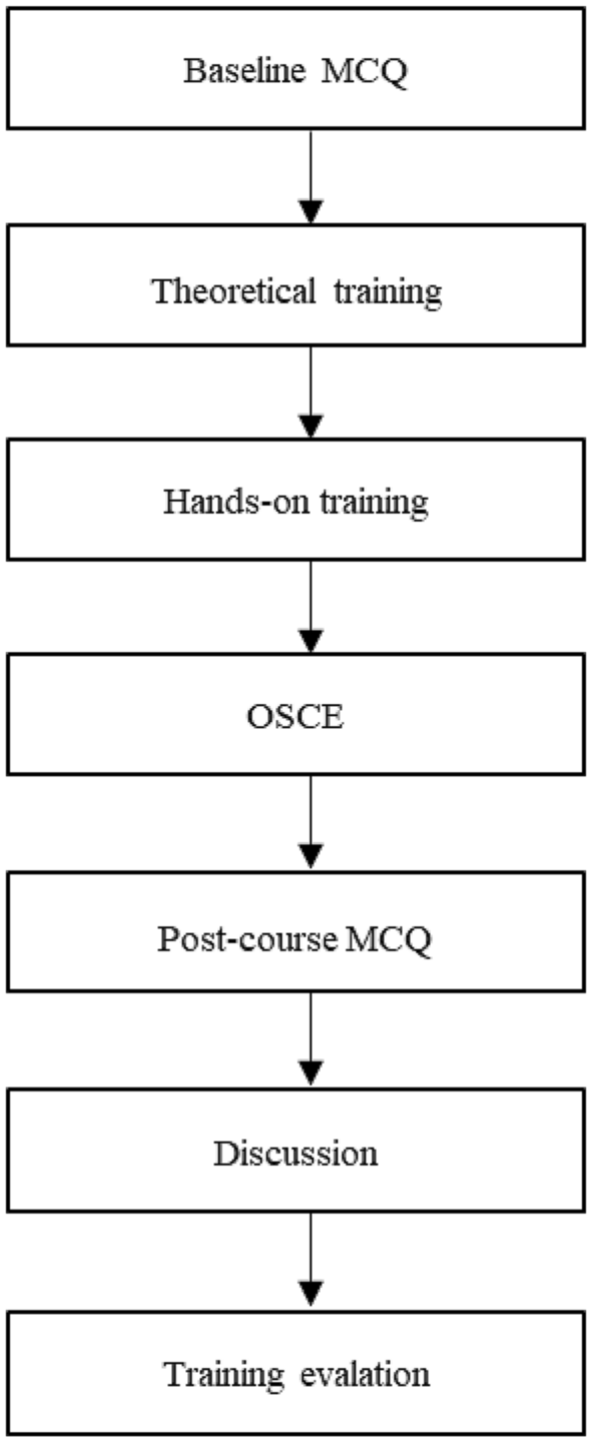
Training program

The training program encompassed 1 h of theoretical and 3 h of hands-on training. Prior to starting both training sessions, the student’s knowledge was assessed with a baseline test of 15 multiple choice questions (MCQ). The increase in knowledge was tested approx. 5 h afterwards applying the same set of MCQ again. However, the answers have not been discussed and reviewed after the initial test so that the difference pre- vs. post-training reflected the gain in knowledge achieved in the training. In addition, an “objective structured clinical examination” (OSCE) and a “student curriculum evaluation” were applied. (Fig. 1.).

In the theoretical session, students learned basics on breast US examination, in particular US features of benign and malignant lesions including the International Breast Imaging Reporting and Data System classification (BIRADS) [15].

**Fig. 2:**
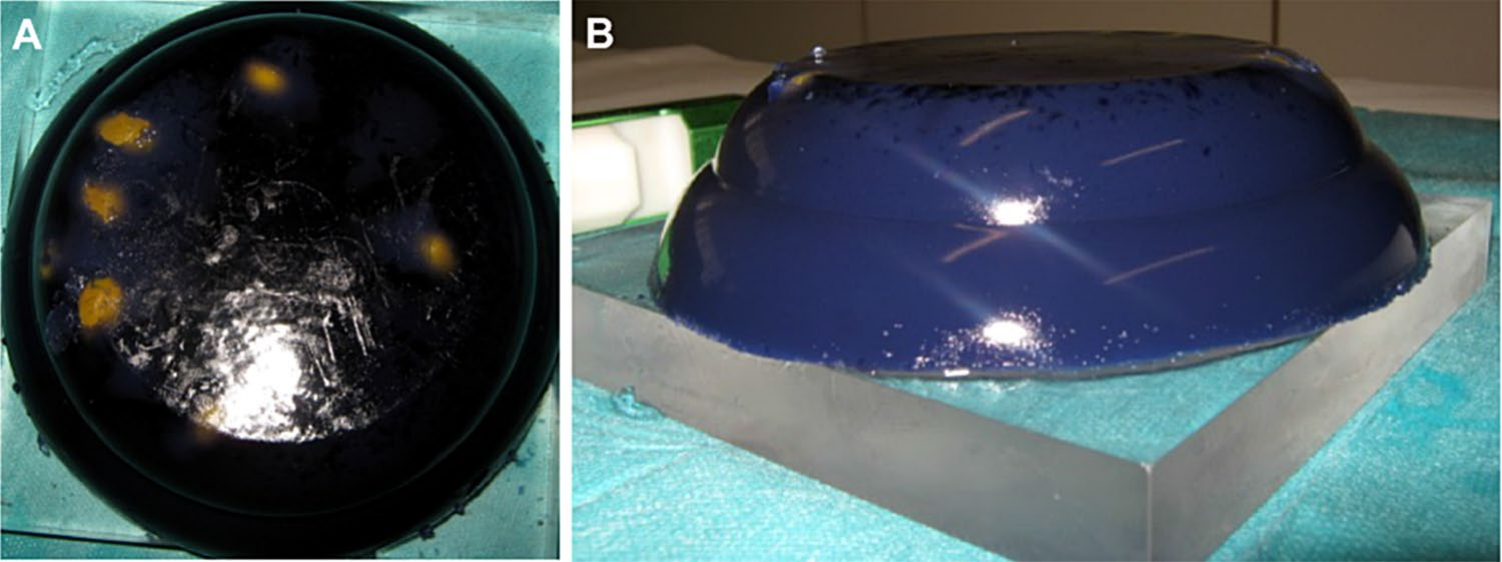
Training phantom made of agar–agar **a** Viewed from above (floating olives yellowish); **b** Viewed from the side

**Fig. 3:**
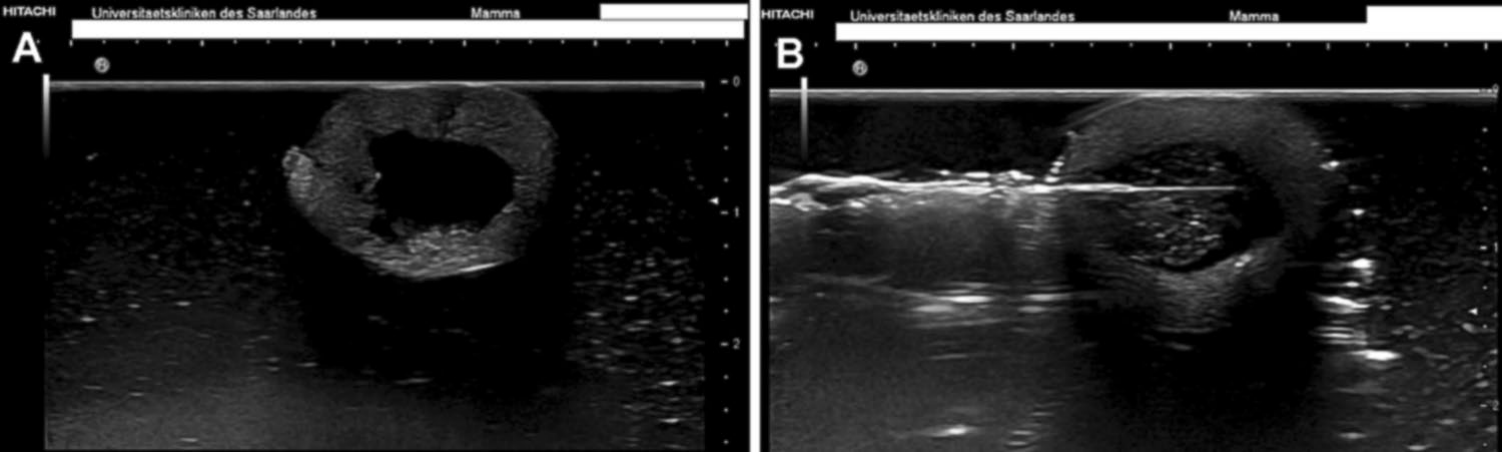
Ultrasound image of training phantom with embedded green olives. **a** Before; **b** After core needle biopsy

In the hands-on session, students were instructed in the use of the US device for breast screening in patients and how to perform a US-guided core-needle biopsy on the training phantom (Figs. 2, 3).

The hands-on session applied Peyton’s 4-step training approach for skills teaching [16], as reported Hamza A et al. [11]. These four steps were: Step 1: “Demonstrate”: The trainer demonstrates the skill at a normal pace and without additional comments; Step 2: “Talk the trainee through”: the trainer demonstrates the respective skill while describing each procedural sub-step in detail; Step 3: “Trainee talks trainer through”: the trainer performs the skill for a third time, based on the sub-steps described to him by the trainee; Step 4: “Trainee does”: the trainee performs the skill on his/her own.

The whole training program focused on the following skills: (1) DEGUM protocols for breast US screening; (2) scanning technique; (3) selection the correct US probe; (4) application of gel on patient; (5) adjustment of the image depth or contrast to optimize the visualization of the lesion; (6) setting of the focus point; (7) correct measurement of lesions; (8) freezing and printing the image; (9) visualization of core needle tip throughout the whole procedure; and (10) advancement of core needle tip into the lesion.

### Training phantom and US devices

Students practiced the US-guided core-needle biopsy on an in-house-designed training phantom which looked like a “round pie” of black ink colored agar–agar gel, with several green olives floating inside, mimicking breast lesions (Fig. 2). Presence of olive material inside the biopsy needle confirmed a correct biopsy.

The US devices were Hitachi Preirus and Hitachi Ascendus. The biopsies were performed using BARD^®^ MAGNUM^®^ biopsy system.

### Knowledge evaluation

#### MCQ

A total of 15 multiple choice questions (MCQ) were picked from the UCAN (Umbrella Consortium for Assessment Networks) questions pool about senology and breast US [17]. There were 12 questions on scientific background and three on image recognition.

For each single question, the null hypothesis that the proportion of right answers was equal before and after the course was tested using the Chi-square test. A summary score of the number of right answers was calculated per student and per time point. The null hypothesis that the total number of right answers was equal before and after the course was tested using the nonparametric Kruskal–Wallis test.

As appropriate for explorative analyses, a comparison-wise two-sided significance level of 5% was used. The statistical analyses were performed using the package arsenal of the R (r-project.org) software.

### Objective structured clinical examination (OSCE)

All students passed the Objective Structured Clinical Examination (OSCE). They were asked to examine an “imaginary subject” on two stations. On the “Ultrasound Station” they had to take the (1) medical history and (2) perform a clinical and ultrasound breast examination. On the “Punch Station” they had to obtain (3) a patient consent form and (4) perform an US-controlled core-needle biopsy on the agar–agar phantom. For each of the four categories 4–6 pre-defined requirements were scored with points. The maximum was 20 points.

### Student curriculum evaluation

At the end of the training, the students were asked to evaluate the program using a standardized five-point scale questionnaire (1 = very good, 2 = good, 3 = satisfying, 4 = adequate, 5 = inadequate) on 20 questions (Table 3).

## Results

A total of 40 students took part in the training program.

### MCQ

The MCQ score increased significantly during the training from 7.5 to 11.3 (*p* < 0.001), as did the percentage of correct answers (50.17–75.17%, *p* < 0.001) (Table 1).

**Table 1:** MCQ assessment before and after the training

MCQ	Before	After	*P* value
Mean score (range)	7.5 (2–12)	11.3 (5–15)	< 0.001
Percentage (%) correct answers	50.17%	75.17%	< 0.001

**Fig. 4:**
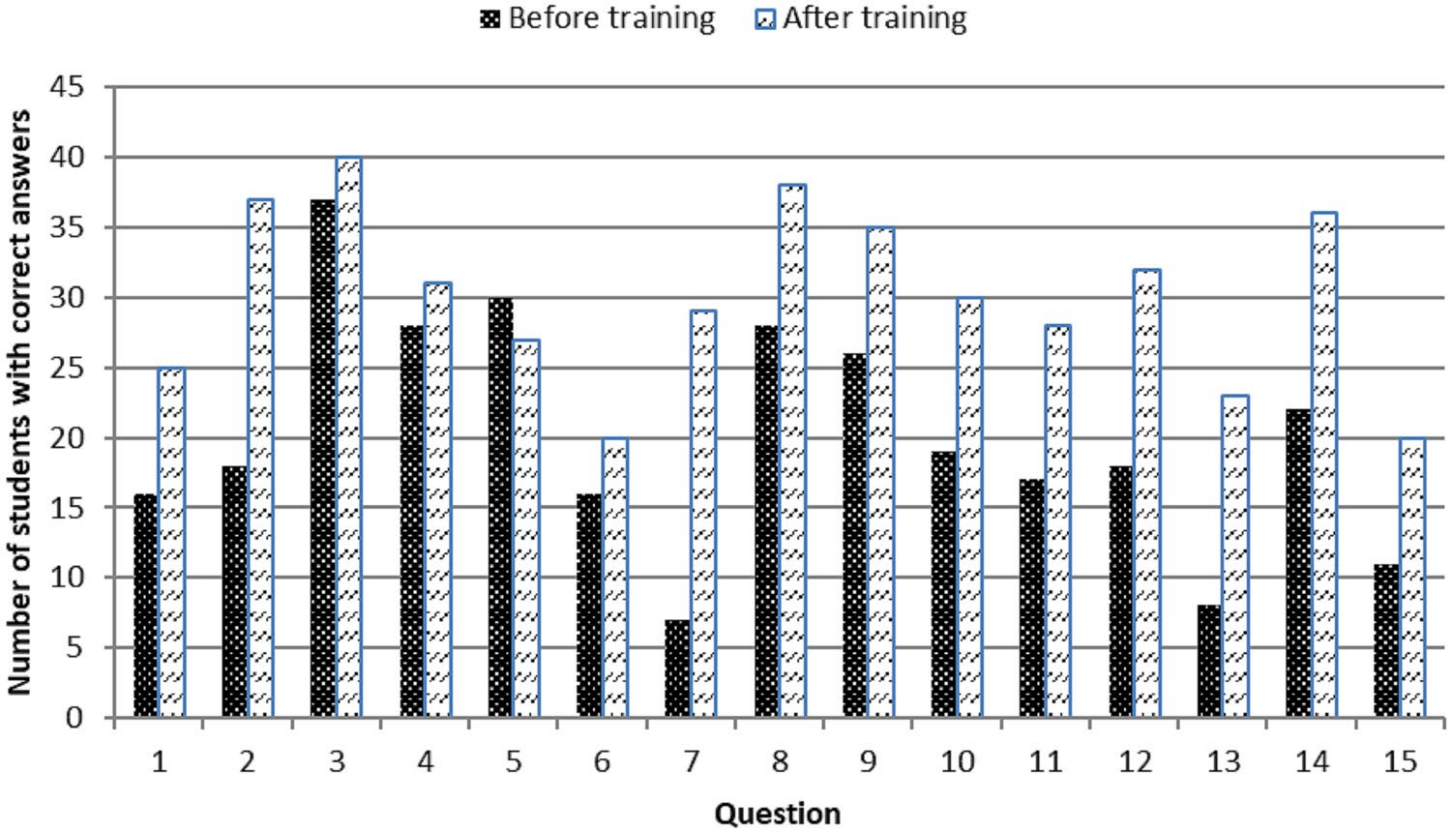
Number of students with correct answers to 15 MCQs, before and after the training program

Except for question #5, the number of students with correct answers increased after the training (Fig. 4).

### OSCE

The results of the OSCE are shown in Table 2. The mean total was 17.3/20 points (86.5%), confirming the implementation of the new skills. The 4 categories (medical history, breast US, patient consent form and core-needle biopsy) were assessed individually. Students scored highest in patient’s medical history (97.5%) and lowest for core needle biopsy. All scores were ≥ 80%) (Table 2).

**Table 2:** OSCE results after the training (*n* = 40)

	Achieved score	Max. possible score	%
Patients medical history	3.9	4	97.50%
Breast US screening	5.1	6	85.00%
Patient consent form	3.5	4	87.50%
Core needle biopsy	4.8	6	80.00%
Total	17.3	20	86.50%

### Student curriculum evaluation

The student curriculum evaluation in general was very positive. A total of 16/20 questions were rated between 1.2 and 1.7 (very good) and three questions were rated 2.1 s (good).

Only one question about the requirements of the training program was rated as satisfying (3.0).

The most crucial questions “How do you rate the learning success of this program?”, “Overall, how do you rate the program?” and “Would you recommend the program?” were very positive. All questions and liker scores are shown in Table 3.

**Table 3:** Student curriculum evaluation questions expressed by mean liker scores

Question	Mean
How well were the stated learning objectives defined?	1.5
How do you rate the quality of the classroom and the technical equipment?	1.4
How do you rate the punctuality and regularity of the course?	1.4
To what extent was the course understandable and clearly processed?	1.5
To what extent were medical/clinical references established?	1.3
To what extent were references made to current topics?	1.7
To what extent do you value the course as relevant to the exam?	2.1
How do you rate the quality of the teaching materials?	1.6
Were suggestions given for in-depth study?	2.1
How do you value the professional competence of the instructor?	1.2
How did you perceive the learning and working atmosphere?	1.5
How do you rate the motivation and preparation of the instructor?	1.2
Could you follow the instructor well?	1.4
Did the instructor repeat the content adequately?	1.3
How would you rate the opportunity to ask questions and the willingness to discuss?	1.3
How good was interdisciplinary teaching?	2.1
How do you rate the learning success of this course?	1.5
Overall, how do you rate the course?	1.4
How do you rate the requirements of the course for yourself?	3.0
Would you recommend the course?	1.4

Liker scores: 1 very good, 2 good, 3 satisfying, 4 adequate, 5 inadequate

## Discussion

This study assessed the effectiveness of an extended US breast training program on the knowledge and hands-on skills of undergraduate medical students. Students’ knowledge increased, and students’ satisfaction was high on completion of the course.

The results of the MCQ and the OSCE clearly showed that this supplemental training program provided value. The positive individual perception of the training was shown in the results of the student curriculum evaluation. Students particularly enjoyed the hands-on training part, using the US probe and performing core-needle biopsies on the phantom. They would recommend this program and similar learning opportunities to their fellow students.

Some other groups have reported on their experience in teaching US skills and US-guided core-needle biopsy to undergraduates. Limchareon S et al. showed the beneficial effect of a two-week rotation training in radiology on 48 student’s US skills, regardless of their baseline performances. They also used the OSCE as a standardized assessment tool [18].

Ault et al. performed a breast workshop focusing on physical examination, mammography and US interpretation. They stated that this workshop was more effective than the traditional outpatient setting for teaching clinical breast examination skills [19].

Hamza et al. [11] and Takacs et al. [20] reported similar positive results from comparable programs in other disciplines.

In this study an agar–agar training phantom was used for the core-needle biopsies. This is highly cost-effective (approx. 2 Euros per phantom) and allowed avoiding usage of fresh cadavers as proposed by McCrary H et al. [13] or fleshy tissue like turkey breast [21]. However, all these training models are for single use only. Another, more expensive option for US breast teaching purposes including biopsies is mannequin simulators. These simulators were already used for teaching obstetrical US by Chalouhi et al., who reported no differences between training results on the mannequin versus pregnant volunteers [22].

Young physician’s education is key for the future of healthcare systems in order to offer highest standards in diagnosis and treatment. Currently, an update of undergraduate teaching is being developed in Germany (Masterplan 2020) with increased focus on hands-on trainings of medical students [23]. This will certainly require more teaching facilities. This study provides additional evidence for the effectiveness of this new teaching approach.

Some limitations need to be addressed: (1) The training phantom made of agar–agar does not realistically resemble the human breast; (2) The phantom can only be used for one training session; (3) This training program required a large time commitment from the teaching personal, and because of staff and budget constraints in many hospitals, might not yet be feasible in the routine setting [11].

## Conclusion

Undergraduate student’s medical education can be enhanced by teaching breast US skills.
